# Penicillin and Oxacillin Loaded on PEGylated-Graphene Oxide to Enhance the Activity of the Antibiotics against Methicillin-Resistant *Staphylococcus aureus*

**DOI:** 10.3390/pharmaceutics14102049

**Published:** 2022-09-26

**Authors:** Mohadeseh Mohammadi Tabar, Moj Khaleghi, Elham Bidram, Atefeh Zarepour, Ali Zarrabi

**Affiliations:** 1 Department of Biology, Faculty of Sciences, Shahid Bahonar University of Kerman, Kerman 76169-14111, Iran; 2 Biosensor Research Center, School of Advanced Technologies in Medicine, Isfahan University of Medical Sciences, Isfahan 81746-73461, Iran; 3 Department of Biomaterials, Nanotechnology and Tissue Engineering, School of Advanced Technologies in Medicine, Isfahan University of Medical Sciences, Isfahan 81746-73461, Iran; 4 Department of Biomedical Engineering, Faculty of Engineering and Natural Sciences, Istinye University, Istanbul 34396, Turkey

**Keywords:** graphene oxide, antibiotic resistance, penicillin, oxacillin, methicillin-resistant *Staphylococcus aureus*

## Abstract

Infectious diseases are known as the second biggest cause of death worldwide, due to the development of antibiotic resistance. To overcome this problem, nanotechnology offers some promising approaches, such as drug delivery systems that can enhance drug efficiency. Herein, a Graphene Oxide-polyethylene glycol (GO-PEG) nano-platform was synthesized and penicillin and oxacillin, two antibiotics that are ineffective against Methicillin-resistant *S. aureus* (MRSA), were loaded on it to improve their effectiveness. The nanocomposites were characterized using FTIR, XRD, UV–Vis, FE-SEM/EDX, and Zeta potential analyses, followed by an evaluation of their antibacterial activity toward MRSA. Based on the results, drug loaded GO-PEG nanocomposites with loading efficiencies of 81% and 92% for penicillin and oxacillin, respectively, were successfully synthesized. They showed a controlled release within six days. The zeta potential of GO-PEG-oxacillin and penicillin was −13 mV and −11 mV, respectively. The composites showed much more activity against MRSA (80–85% inhibition) in comparison to GO-PEG (almost 0% inhibition) and pure antibiotics (40–45% inhibition). SEM images of MRSA treated with GO-PEG-antibiotics showed a deformation in the structure of bacterial cells, which led to the collapse of their intracellular components. These results demonstrate the effectiveness of utilizing the GO-based nanoplatforms in enhancing the antibacterial activity of the antibiotics.

## 1. Introduction

The control of infectious diseases has long been one of the most challenging issues because they are usually caused by multidrug-resistant microorganisms, which results in long-term illness and increased risk of death [1]. Antibiotics are among the essential drugs that have attracted much attention due to their application in the treatment of bacterial infections. However, treating severe infections has become more complicated due to the abuse of antibiotics [2,3]. The overuse and misuse of antibiotics has caused the number of antibiotic-resistant bacteria to rise and has led to the persistence of these microorganisms [4]. Methicillin-resistant *Staphylococcus aureus* (MRSA), discovered in 1961, is considered to be one of the most critical multi-drug-resistant pathogens and plays a substantial role in increasing various infections in different communities, so much so that MRSA strains are now endemic in hospitals worldwide [5]. This strain shows resistance toward beta-lactam antibiotics, including penicillin and methicillin, and leads to significant clinical treatment problems [6]. Currently, MRSA infections are treated with non-β-lactam antibiotics, such as clindamycin, which in turn results in more resistant strains [7]. The new global priority pathogens list (PPL) of antibiotic-resistant bacteria published by the WHO recommends that further studies are necessary to discover effective ways to overcome these bacterial species [8].

**Figure 1:**
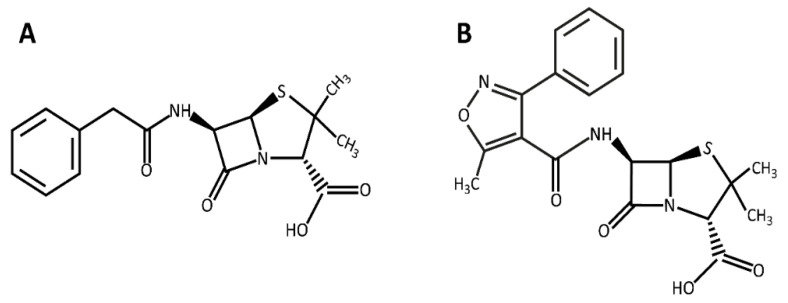
Chemical Structure of (**A**) penicillin and (**B**) oxacillin.

Benzylpenicillin or penicillin G (PEN; Figure 1A) belongs to the β-lactam antibiotics with the main structure of the thiazolidinedione ring, discovered by Fleming in 1928 [9], and was initially introduced as the primary treatment for bacterial contagions [10,11]. Soon after introducing penicillin, resistance against this antibiotic was observed among *S. aureus* strains. These strains produce and secrete a β-lactamase enzyme that destroys the β-lactam ring [12,13]. Sodium oxacillin (5-methyl-3-phenyl-4-isoxazolyl penicillin sodium; Figure 1B) primarily targets the penicillin-binding protein 2 (PBP2) and prevents bacterial cell wall synthesis [14,15]. Other classes of antibiotics, which are currently effective on MRSA, such as vancomycin, aminoglycosides, and macrolides, are found to be useless due to antibiotic resistance development [16,17,18]. Therefore, finding a rational strategy to enhance the antibacterial capability of the existing antibiotics, and thus overcome the bacteria resistance, is a crucial issue that has recently become one of the most important topics in biological research worldwide.

At present, nanoscience and technology have introduced novel concepts of material sciences, which has resulted in promising achievements in all research fields. In biomedicine especially, this technology comes up with many solutions to overcome existing limitations [19,20]. The design and development of nano-carriers to smart drug delivery are the most interesting research topics that have recently had striking achievements [21,22]. Due to their natural properties, nanostructures can be combined with biologically active substances or other nanomaterials and can be used as effective drug carriers and diagnostic agents. Thus, nanoplatforms could potentially be used in drug delivery, biosensors and bioimaging, antibacterial agents, protective coating, tissue engineering, photothermal therapy, etc. Nanoplatforms, as vehicles, enable more effective and targeted action on a specific tissue or pathogen, which results in less toxicity to the whole organism [23,24].

Graphene oxide (GO) is one of the common types of two-dimensional nanomaterials with several hydroxyls, carboxyl, and epoxy functional groups with good water solubility and biocompatibility. The presence of these functional groups makes it possible to attach different functionalizing agents to the surface of GO and improve its features. This option facilitates the utilization of GO for several purposes, such as in drug/gene delivery systems, antibacterial agents, biosensors, and water purity [25,26]. It has been revealed that GO sheets can affect the cell membrane and ultimately destroy bacterial cells [27]. Furthermore, it has been demonstrated that GO can make strong interactions with organic compounds, making it a more suitable drug carrier to counter pathogens [28]. For instance, Yang Gao et al. loaded three antibiotics (lincomycin hydrochloride, chloramphenicol, and gentamicin sulfate) on GO and investigated their activity against *E. coli* and *S. aureus*. It was revealed that GO coated and damaged bacteria cell membranes, which inhibited bacterial growth. Deactivation of *E. coli* and *S. aureus* with GO was dependent on concentration and time [28].

To improve the GO performance, it can be chemically modified and composited with polymers/biopolymers, biomolecules, and inorganic nanomaterials [5]. Polyethylene glycol (PEG), for example, has various applications in the biological, chemical, and pharmaceutical fields due to its desirable properties such as flexibility, hydrophilicity, and biosafety [29]. This polymer plays a central role in drug delivery systems as well [30]. Decoration of GO with PEG increases the water solubility and biocompatibility of GO. It also allows for the possibility that some parts of therapeutic agents can be entrapped between the hydrophobic parts of the polymer, which increases the loading capacity of GO. The presence of PEG can also control the release rate of drug components in a sustainable manner [31].

According to the aforementioned features, this study aimed to evaluate the effect of utilizing GO-PEG on reviving and enhancing the performance of antibiotics against Methicillin-resistant *S. aureus.* To reach this aim, PEGylated GO composites loaded with two different types of β-lactam antibiotics, Penicillin (PEN) and Oxacillin (OXA), were synthesized and characterized with different types of analytical tests, such as FTIR, XRD, UV–Vis, FE-SEM/EDX, and zeta potential analysis. The loading and release pattern of the drug-loaded nanocomposites were then evaluated, and their antibacterial activity was assessed against the Methicillin-resistant *Staphylococcus*.

## 2. Materials and Methods

### 2.1. Materials

GO was obtained from Graphene X Co., Tehran, Iran. Penicillin G sodium salt (CAS 69-57-8), oxacillin sodium salt (CAS 7240-38-2), and 1-ethyl-3-(3-dimethylamino propyl) carbodiimide hydrochloride (EDC) were purchased from Sigma-Aldrich Chemical Co. (St. Louis, MO, USA). Polyethylene glycol (PEG_6000_) was bought from Carl Roth Co. (Karlsruhe, Germany). 4-dimethyl aminopyridine (DMAP) was purchased from Merck (Darmstadt, Germany). MH (Muller-Hinton) agar medium was purchased from Scharlau Co. (Barcelona, Spain). The MRSA ATCC 33591 bacterial strain was purchased from the Pasteur Institute of Iran, Tehran, Iran.

### 2.2. Methods

#### 2.2.1. PEGylation of GO

To provide GO-PEG, 0.1 g GO was dissolved in 100 mL deionized (DI) water, and after 2 h of sonication, 0.3 g EDC and 0.2 g DMAP were solved in 100 mL DI water and added to the mixture.

Next, 0.2 g PEG mixed with 50 mL DI water was added to the GO mixture, and it was heated at 60 °C for two days via a heater stirrer. After that, the synthesized GO-PEG was dialyzed (12,000 Da cutoff) against DI water for 24 h, during which the external solution was replaced three times to remove the impurities. Finally, the product was centrifuged and freeze-dried (Vaco 5-ZIRBUS Tech; Bad Grund, Germany) for future use [32].

#### 2.2.2. The Antibiotic Loading on GO-PEG

To achieve the highest amount of each antibiotic that can be loaded on GO-PEG, the percentage of the antibiotics loaded on the GO-PEG platform was evaluated regarding two different ratios of the antibiotic: GO-PEG during 6 h and 24 h. Briefly, the antibiotics solutions were prepared by adding 6 mg of PEN or OXA into 6 mL DI water to get a 1 mg/mL solution. Afterward, the antibiotic solutions were mixed with 3 mL and 6 mL of the prepared GO-PEG solutions to provide 2:1 and 1:1 (*v*/*v*) ratios, respectively. The final mixtures were wrapped with an aluminum foil to prevent the possible photo-degradation of the antibiotics and moderately shaken for 6 and 24 h at 4 °C. The solutions were then filtered, and the concentrations of the residual antibiotics in the solutions were determined using the calibration curves obtained by evaluating their UV–Vis absorbance at the wavelength of 273 nm and 204 nm for OXA and PEN, respectively. The percentage of the antibiotic loading capacity (LC) was calculated based on Equation (1) [28,33].

LC (%) = (Total mass of loaded drug/Total mass of nanocarrier) × 100(1)

#### 2.2.3. Characterization

The UV–Vis spectra of GO-PEG and GO-PEG-antibiotics were recorded from 200 to 800 nm (Jenway 7315 Spectrophotometer; Cambridge, UK) to confirm the formation of the compounds. The surface functional groups of GO-PEG and GO-PEG-antibiotics were studied using Fourier-transform infrared spectroscopy (FTIR, JASCO 6300; Tokyo, Japan). X-ray crystallography (XRD, Asenware AW-DX300, R&D and supply chain center Zhongshan Guta Fire equipment technology Co., Ltd, Guangdong, China) was used to investigate the GO-PEG crystallographic features using Cu Ka radiation, and 2θ = 5–80°. The Field Emission Scanning Electron Microscope (FESEM, Quanta 450 FEG; CA, USA) was used to study the GO-PEG and GO-PEG-antibiotics morphology. The chemical composition of GO-PEG-antibiotics was also studied using EDX analysis coupled with FE-SEM. The zeta potential of the compounds was evaluated by Horiba SZ-100 particle size analyzer (Horiba SZ-100; Kyoto, Japan) [34,35,36,37].

#### 2.2.4. Drug Release Evaluation

The release mode of the antibiotics from the GO-PEG nanocarrier during the time was investigated in PBS at the physiologic temperature (37 °C). For this purpose, 0.5 mg of each GO-PEG-antibiotic compound was dispersed in 2 mL PBS buffer (pH 7.4) and incubated in a shaker incubator (180 rpm) at 37 °C. After different interval times (2, 4, 8, 12, 24, 48, 72, 96, and 120 h), the whole samples were taken and centrifuged. The concentration of the antibiotics released into the supernatant was measured using UV–Vis at the wavelength of 205 nm and 273 nm for penicillin and oxacillin, respectively. The percentage of released antibiotics was calculated using Equation (2) [37].

Drug release (%) = (Concentration of released drug/Concentration of loaded drug) × 100 (2)

#### 2.2.5. The Evaluation of the Antibacterial Activity

##### Broth Microdilution Method

To determine the minimum inhibitory concentration (MIC) of the GO-PEG-OXA and GO-PEG-PEN, their solutions were provided in the range of 25 μg/mL to 600 μg/mL. Then, 100 μL of each solution was added to the wells of a 96-well plate already filled with 150 μL of the bacterial suspension (0.5 McFarland). Wells containing untreated bacteria were considered controls. After 18 h of incubation at 37 °C, to assess the survival of MRSA exposed to the compounds, the optical density (OD_600_) of each well was measured using a microplate reader (Bio-Rad Laboratories, Inc., Hercules, CA, USA). Finally, the bacterial cell growth rate was determined according to Equation (3).

Bacterial growth (%) = ((OD control − OD sample)/OD control) × 100(3)

To determine the minimum bactericidal concentration (MBC), wells that showed no visible turbidity were transferred to the MH agar plates and incubated at 37 °C for 24 h. The minimum concentration at which no bacterial cell grew was considered as MBC [38,39].

##### FE-SEM Analysis

For a more detailed study, the interaction of the GO-PEG-antibiotic components with bacterial cells was investigated via FE-SEM. For this purpose, an overnight bacterial culture was provided and adjusted to the 0.5 McFarland standard. Next, 100 μL of this bacterial suspension was transferred to the wells of a 6-well plate. Then, 1 mL of each nanocomposite solution (100 μg/mL in uncultured medium) was added to the wells, a sterile lamel was embedded into each one, and the pleats were incubated for 18 h at 37 °C. After that, the lamels were taken out, washed three times with PBS, and sunk in Glutaraldehyde (3%) for 30 min. After this time, the lamels were washed again and immersed in ethanol with concentrations of 30, 50, 70, 90, and 100%, respectively (10 min in each one) and analyzed by FE-SEM after overnight drying under a laminar hood [40].

#### 2.2.6. Statistical Analysis

Data were quantitative analysis by SPSS software (version 21, parametric analysis of variance [ANOVA (Tukey)], IBM; Chicago, IL, USA) and the results are reported as mean values ± standard deviation (SD). *p* ≤ 0.05 was selected as significand.

## 3. Results and Discussion

### 3.1. PEGylated GO Characters

To increase the biocompatibility and solubility of the proposed nanoplatforms, GO was modified with PEG polymer using EDC and DMAP as intermediates. The FTIR spectra of GO, PEG, and GO-PEG are presented as Figure S1A. According to the FTIR spectrum of GO, the relatively broad and intense peak that appeared in the region around ~3000 to 3700 cm^−1^ is attributed to the stretching vibration of the hydroxyl groups [41]. The ~1720 cm^−1^ peak is relevant to the C=O band of the carbonyl groups [42,43]. Two peaks that appeared at ~2847 cm^−1^ and 1104 cm^−1^ in the PEG spectrum can be attributed to C-H and C–O groups, respectively [44]. Peaks that appeared at 1468–1342 cm^−1^, 1280–1242 cm^−1^, and 1149 cm^−1^ were attributed to the deformation vibration of C–H, bending vibration of O–H, and stretching vibration of C–O, respectively [45]. Considering the FTIR spectrum of GO-PEG, the stretching band of O–H and carboxyl groups of GO and PEG were noticeable, and the absorption bands of the main functional groups originating from PEG are detected with only a trivial shift in the position and relative intensity of the peaks. The observation of the peaks of functional groups of PEG confirms the successful conjugation of GO nanosheets with PEG [46]. 

Crystallographic structures of nanoformulations after each modification were conducted via XRD spectroscopy and the results are shown in Figure S1B. We saw in the figure that the crystallographic peak of GO was located at 2θ = 10.4°, which is related to the (001) crystalline plane [47]. After modification of the surface of GO with PEG, a wide peak appears at 2θ = 10–20°, which is normally considered an amorphous peak and could indicate that the composite of GO-PEG has an amorphous structure [37,47]. This could also be confirmed by comparing the XRD patterns of PEG and GO-PEG; however, some characteristics of the peaks of PEG have remained in the GO-PEG curve [43].

**Figure 2:**
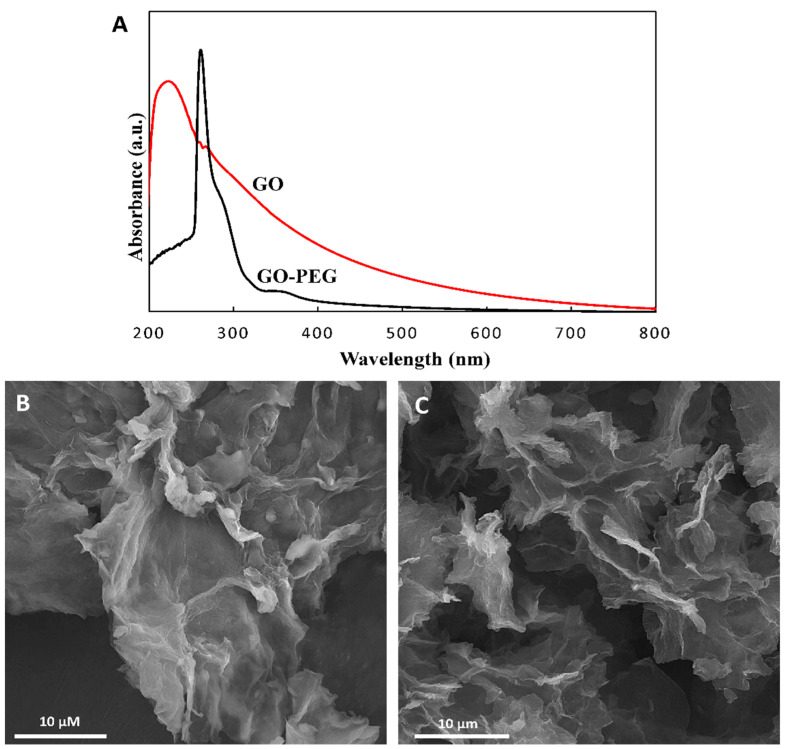
(**A**) UV–Vis spectra of GO and GO-PEG; (**B**) The FE-SEM images of GO and (**C**) The FE-SEM images of GO-PEG.

The UV–Vis spectra of GO and GO-PEG confirmed the success of the modification (Figure 2A). The detected peak at 233 nm is typical of pure GO and determines the degree of remaining conjugation (π–π* transition) in GO. The shoulder around 280–290 nm can be attributed to the n–π* transition of carbonyl groups [48]. The shift of the GO peak at 233 nm to 280 nm after coating with PEG indicates successful surface modification.

The morphology of GO and GO-PEG was studied by FE-SEM analysis. Results indicated a GO layer with thin and sharp edges and flat surfaces (Figure 2B). In contrast with GO, the GO-PEG structure appeared to have thicker and wrinkled plates, as shown in Figure 2C. The thick and twisted structure could confirm PEG attachment on the GO surface. Another difference between GO and GO-PEG is the difference in their thickness. When PEG is exposed and attached to GO nanosheets, it leads to separation, so the FE-SEM image of the nanosheets has less thickness. In GO samples, different layers are stuck together, and this sample had more thickness in FE-SEM images.

### 3.2. The Loading of Antibiotics on the GO-PEG

Table 1 shows the results of drug loading efficiency on GO-PEG. Based on the results, the best drug loading was observed for the 2:1 ratio, at the reaction time of 6 h.

### 3.3. GO-PEG-Antibiotics Characters

**Figure 3:**
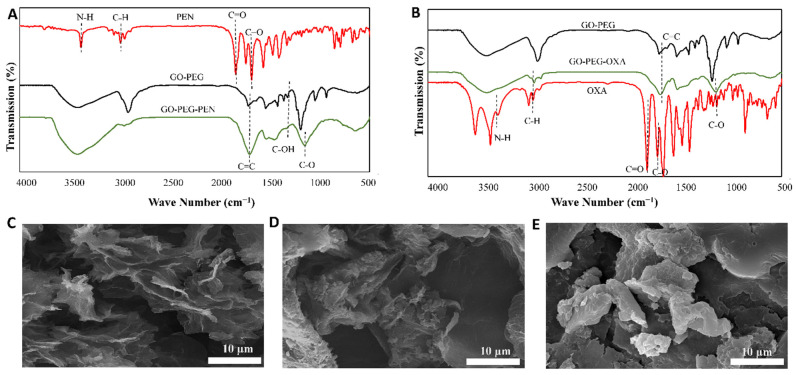
FT-IR spectra of (**A**) GO-PEG-PEN (Penicillin (red), GO-PEG (black), and GO-PEG-penicillin (green)); (**B**) GO-PEG-OXA (Oxacillin (red), GO-PEG (black), and GO-PEG-oxacillin (green)); and the FE-SEM images of (**C**) GO-PEG without antibiotic; (**D**) GO-PEG-PEN, and (**E**) GO-PEG-OXA.

The surface functional groups of the designed compounds (GO-PEG-PEN, GO-PEG-OXA) were investigated using the FTIR (Figure 3A,B). Since PEN and OXA are from the same family (beta-lactam antibiotics), they have relatively similar structural and functional groups. For both, the characteristic bands appear at 3384 cm^−1^ (N-H) and 1680 cm^−1^ (C=O) [49,50]. The peaks that appear at 1700 cm^−1^ and 2910 cm^−1^ correspond to the carboxyl functional groups (COOH) and C-H groups, respectively [51,52]. In GO-PEG-PEN and GO-PEG-OXA, the shifting of the PEN and OXA absorption bands to ~2900 cm^−1^ (C-H), 3400–3590 cm^−1^ (N-H), 1610–1620 cm^−1^ (C=C), and 1654 cm^−1^ (C=O), as well as the appearance of the new bands at 1220 cm^−1^ (C-OH) and 1045 cm^−1^ (C-O), indicate the introduction of oxygen-containing groups into the graphene surface. The peak detected at the 1600 cm^−1^ represents the cyclic alkenes (C=C) in the OXA structure [27,53,54].

Furthermore, the different morphology of GO-PEG-PEN and GO-PEG-OXA compared to GO-PEG indicates the presence of antibiotics inside the GO-PEG surface (Figure 3C–E).

**Figure 4:**
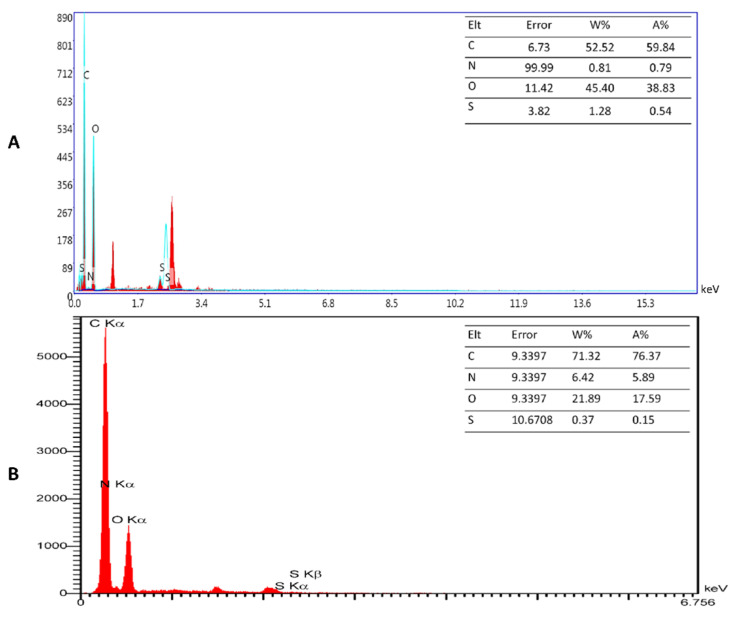
The EDX spectra of GO-PEG-PEN (**A**) and GO-PEG-OXA (**B**).

In addition, the chemical composition of GO-PEG-PEN and GO-PEG-OXA were evaluated using EDX analysis (Figures S2 and S3). The obtained EDX-maps revealed that the main elements, such as carbon (C), oxygen (O), nitrogen (N), and sulfur (S), were distributed on the drug-containing platforms in a monodisperse manner. As Figure 4 shows, the EDX point analysis provided the specific percentage of the elements in each composite. Table 2 also shows the full data of EDX analysis. The data presented in this table are average data from three replicates.

**Figure 5:**
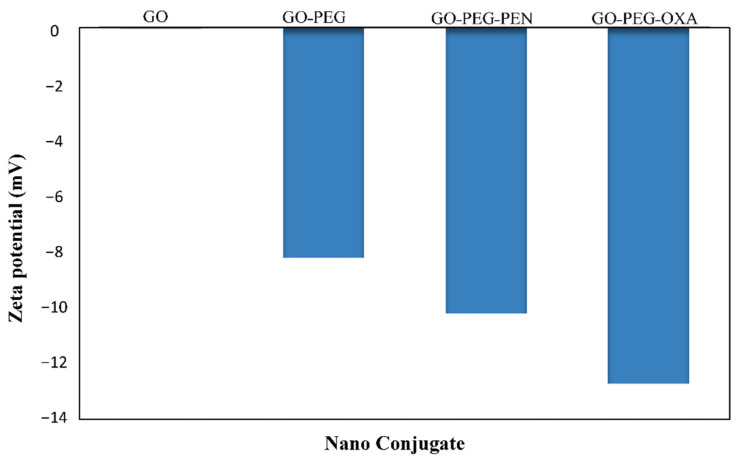
Zeta potentials of GO, GO-PEG, GO-PEG-PEN, and GO-PEG-OXA.

Concerning the compounds’ surface charge, the zeta analysis confirmed that the surface charges of all compounds were negative (Figure 5). This feature was −13, −11, −8, and −0.2 mV for GO-PEG-OXA, GO-PEG-PEN, GO-PEG, and GO, respectively. This negative charge can be attributed to the presence of hydroxyl and carboxyl groups of compounds [55]. The loading of the antibiotics on the GO-PEG resulted in a more negative charge related to the presence of double-bonded carboxylic acid, sulfur, and oxygen groups in the structure of antibiotics. Electrons in both structures can be adsorbed onto the GO-PEG via non-electroactive interactions, including Van der Waals forces, and H bonding for PEN and π-π binding in OXA, due to its aromatic structure.

### 3.4. Drug Release Evaluation

**Figure 6:**
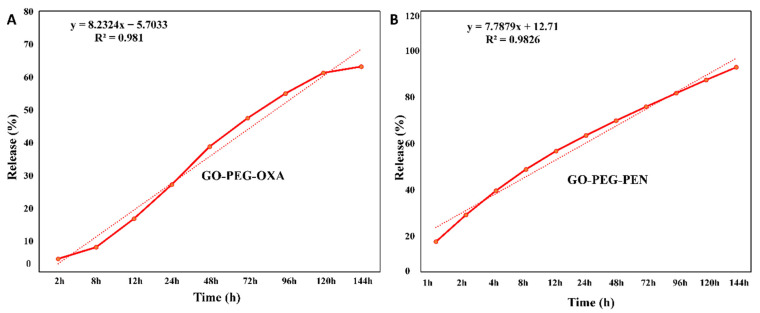
Release of antibiotics from GO-PEG; (**A**) GO-PEG-OXA, (**B**) GO-PEG-PEN.

According to Figure 6, the release of oxacillin and penicillin from the GO-PEG nano-platform was slow, controlled, and increased as time passed. Although both oxacillin and penicillin were released on a gentle gradient, the percentage of penicillin released within six days (95%) was considerably higher than oxacillin (63%). It has been indicated that GO-PEG can absorb antibiotics severely or poorly depending on the number of aromatic rings in their structures and the presence of functional groups [28]. Notably, antibiotics with an aromatic ring, such as OXA, can readily be adsorbed on GO through π–π interactions, while the PEN that has no aromatic ring in its structure can be adsorbed on GO via non-electrostatic interactions, including Van der Waals forces [28]. Based on these factors, the stronger adsorption of OXA on the GO surface in comparison to PEN can justify the lower release rate of this antibiotic.

### 3.5. The Antibacterial Activity Evaluation

**Figure 7:**
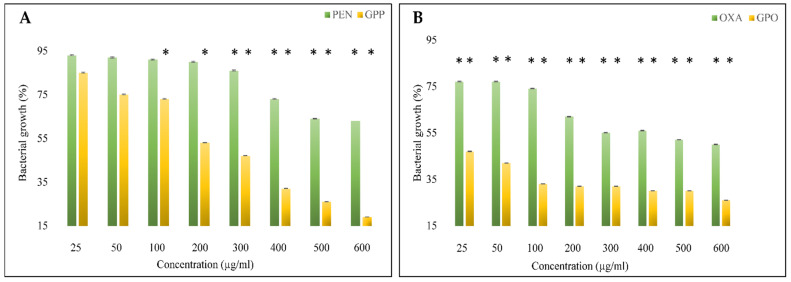
Bacterial growth inhibition of compounds tested against MRSA, (**A**) PEN and GO-PEG-PEN, (**B**) OXA, and GO-PEG-OXA. This chart shows that GO-PEG antibiotics significantly inhibit bacterial growth compared to free antibiotics. (* means *p* ≤ 0.05).

The evaluation of the antibacterial activity of GO-PEG-antibiotics through the microdilution method revealed that the antibacterial activity of free antibiotics was significantly lower than that of GO-PEG-antibiotics at similar concentrations (Figure 7). This activity was dose-dependent concerning all compounds. The data obtained from this test was checked by SPSS software, and the significant data were marked with *. Penicillin was significant at concentration above 300 µg/mL, while GO-PEG-PEN data was considerable at 100 µg/mL and above. In the case of both free and GO-PEG-loaded antibiotics, there was significance from the concentration of 25 µg/mL; however, the antibacterial effects of GO-PEG-OXA were more than double in all samples. These results confirmed the effectiveness of utilizing nanocarriers to improve the antibacterial effects of both antibiotics and the capability of utilizing less doses of drugs.

The bacterial growth inhibition in cells treated with 600 µg/mL of the free penicillin was about 65%. This is when this feature decreased to slightly above 15% when the bacterial cells were treated with GO-PEG-PEN. The free Oxacillin could inhibit 52% of cell growth, while the Oxacillin loaded on GO-PEG inhibited more than 80% of bacterial growth. Although no considerable MBC was determined for these compounds, their MIC against MRSA is presented in Table 3.

**Figure 8:**
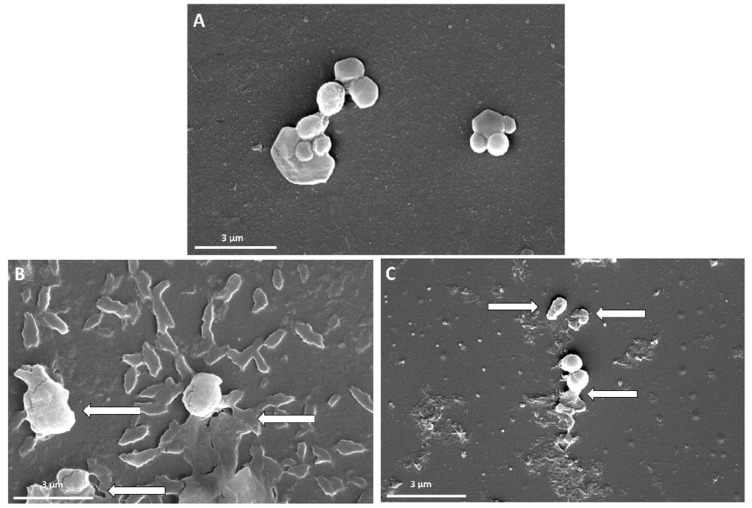
FE-SEM images of MRSA before and after exposure to the GO-PEG-antibiotics, (**A**) MRSA without treatment with GO-PEG-antibiotic, (**B**) MRSA treated with GO-PEG-PEN, (**C**) MRSA with GO-PEG-OXA.

FE-SEM was used to investigate the interactions between GO-PEG-antibiotics and MRSA cells. Figure 8 shows FE-SEM images of MRSA before and after treatment with GO-PEG-PEN or GO-PEG-OXA. According to the results, MRSA cells that were not treated with GO-PEG-antibiotics demonstrated spherical-forming grape-like clusters (Figure 8A), and no parts of the nanosystem were found in it, while exposing bacteria to the drug-loaded nanoformulation led to the deformation of the bacterial membrane (shown by arrows in Figure 8B,C) and consequently killed them. We could also see the nanomaterial components around the bacteria in Figure 8B,C.

Despite the resistance of MRSA to PEN and OXA, the composite of antibiotics and GO-PEG could damage the bacterial cell membranes and increase the destructive power of the antibiotics. The results of our study showed that GO-PEG-antibiotics have antibacterial activity against MRSA, while GO-PEG alone had no antimicrobial effect, which is in agreement with some previous studies [43]. The antibacterial property of GO and GO-PEG were tested, but the info data showed no significant antibacterial properties for the carrier alone (data are not shown).

The results of our study showed that GO-PEG nanosystems can considerably increase the antibacterial activity of antibiotics against MRSA.

There are contradictory reports about the antibacterial activity of GO. However, results of some investigations showed no activity of this compound against bacteria, arguably because the microorganisms preserve their membrane integrity in the face of graphene. It has also been observed that bacteria can grow in the presence of GO and form biofilms [56,57]. In contrast, some studies showed that graphene and its derivatives can inhibit bacterial cell growth [58]. It seems that the biological activity of this carbon material highly depends on its physicochemical properties determined by substances and synthesis procedures. In addition, according to a study conducted by Chen et al., they not only did not see antibacterial effects from graphene oxide, but also claimed that graphene oxide increases the duplication and growth of a set of intestinal microorganisms by acting as a membrane scaffold [59]. Another study was conducted by Ruiz et al., who reported that GO can act as a cell growth promoter for bacterial and mammalian cells [60]. There are different proposed mechanisms for these claims, including that the use of different culture media could lead to the absorption of organic substances from the rich media and thus reduce the antibacterial effect of graphene oxide on it [61]. Moreover, the lack of antibacterial effects of graphene oxide could be due to the reduced level of reactive oxygen species (ROS) in the bacterial biofilm, which can increase bacterial growth [57]. Another important point is the size of graphene oxide, which greatly affects its antibacterial effects [62].

The potential of antibiotics-loaded graphene oxide to combat bacterial resistance has recently been investigated and promising results have been obtained [27,63,64]. The results of a study carried out by Xu et al. show that when vancomycin is conjugated with reduced GO results in a system with a unique antibacterial property against *S. aureus* and *S. epidermidis* [51]. In agreement with previous studies, the results of our research show that GO-PEG-antibiotics have higher toxicity than free antibiotics. This can be related to the increased interaction of GO-loaded antibiotics with the bacterial cell wall, which can lead to an increase in the local concentration of the drug on the bacterial surface and be more effective in damaging cell membranes [28]. GO-PEG could trap bacteria via interacting with their membrane from one side, and directly exposing them with antibiotics from the other side, which could enhance the performance of antibiotics [28]. Another study conducted by Singh et al. used GO as a drug carrier for vancomycin and proved the positive effects of this system on Vancomycin-resistant *Staphylococcus aureus.* The mechanism of action in this system is damaging the cellular membrane by ROS generation and cell lysis [65]. In addition, in another study by Carver et al., the effectiveness of GO with the antibiotic tetracycline on Tetracycline-resistant *Escherichia coli* was tested and inhibited bacterial growth, which confirmed the previous results. They also had used GO-PEG as a carrier for a specific antibiotic and confirmed the effectiveness of this carrier for improving the antibacterial activity of Tetracycline [62] (the same as the current study). Other studies have been conducted in this field that confirmed similar observations [66,67,68].

In this case, it is supposed that the system adheres to the surface of the bacterial cell wall and thus the drug is directly exposed to the bacteria and acts more effectively. Moreover, it has been demonstrated that graphene interacts with biological molecules or cells, depending on the site of interaction, surface, or edges. Due to the limited information on the antibacterial properties of graphene, its mechanisms of action have not been fully explained yet. However, based on the investigations, carbon-based nanostructures can damage bacterial cell membranes and result in oxidative stress (3). These make the bacterial cells vulnerable to antibiotics, which enhances their effectiveness against bacteria.

## 4. Conclusions

We fabricated an efficient nanocomposite based on graphene oxide-polyethylene glycol polymer as a carrier of OXA and PEN antibiotics to combat MRSA antibiotic resistance. The loading of antibiotics on GO-PEG has a significant effect on the antibacterial activity of the compounds. When GO-PEG was loaded with PEN or OXA, their ability to overcome MRSA resistance increased. Results of this study developed an effective nanotechnology-based strategy to improve the therapeutic properties of antibiotics and reduce their shortcomings. However, further research is needed to investigate the interaction of antibiotics with nanoparticles and find appropriate solutions to improve antibiotic performance and reduce MRSA resistance.

## Figures and Tables

**Table 1:** The percentage of drug loading efficiency on GO-PEG in various conditions.

Antibiotics	Ratio & Time			
	2:1		1:1	
	6 h	24 h	6 h	24 h
PEN	81.50	80.37	65.52	61.61
OXA	92.75	84.41	83.28	82.70

**Table 2:** The EDX results for GO-PEG-OXA and GO-PEG-PEN composites.

Sample	Element	Weight (%)				Atomic (%)			
		Min.	Max.	Ave.	STD	Min.	Max.	Ave.	STD
GO-PEG-PEN	C	49.29	52.52	50.5	1.76	55.87	59.84	57.39	2.14
	N	0.81	6.05	4.11	2.87	0.79	5.94	4.03	2.82
	O	42.76	45.4	43.73	1.45	36.77	38.83	37.53	1.13
	S	1.28	1.9	1.67	0.34	0.54	0.82	0.71	0.15
GO-PEG-OXA	C	66.98	71.32	68.72	2.29	73.67	76.37	74.68	1.47
	N	6.42	11.89	9.61	2.85	5.89	11.12	8.98	2.74
	O	16.07	21.89	18.41	3.07	13.16	17.59	15.00	2.31
	S	0.37	5.24	3.26	2.56	0.15	2.16	1.34	1.05

**Table 3:** MIC values of the compounds against MRSA.

Compounds	MIC (µg/mL)
Oxacillin	600 ± 10
Penicillin	700 ± 10
GO-PEG-OXA	100 ± 5
GO-PEG-PEN	200 ± 5

## Data Availability

Not applicable.

## Supplementary Materials

The following supporting information can be downloaded at: https://www.mdpi.com/article/10.3390/pharmaceutics14102049/s1, Figure S1: (A) FT-IR spectra of GO, PEG, and GO-PEG; (B) XRD patterns of GO, PEG, and GO-PEG; Figure S2: EDX mapping of elements (S, O, N, and C) on the GO-PEG-OXA surface. Figure S3. EDX mapping of elements (S, O, N, and C) on the GO-PEG-PEN surface.
