# Supplementary material for: Penicillin and Oxacillin Loaded on PEGylated-Graphene Oxide to Enhance the Activity of the Antibiotics against Methicillin-Resistant Staphylococcus aureus
Source: Pharmaceutics. 2022 Sep 26;14(10):2049. doi: 10.3390/pharmaceutics14102049 (PMC9607092; doi:10.3390/pharmaceutics14102049)
Supplement: Supplementary file 1 [file pharmaceutics-14-02049-s001.zip › pharmaceutics-1918521-supplementary.pdf]

pharmaceutics MDPI

Supplementary Materials: Penicillin and Oxacillin Loaded on PEGylated-Graphene Oxide to Enhance the Activity of the Antibiotics against Methicillin-Resistant *Staphylococcus aureus*

Mohadeseh Mohammadi Tabar, Moj Khaleghi, Elham Bidram, Atefeh Zarepour and Ali Zarrabi

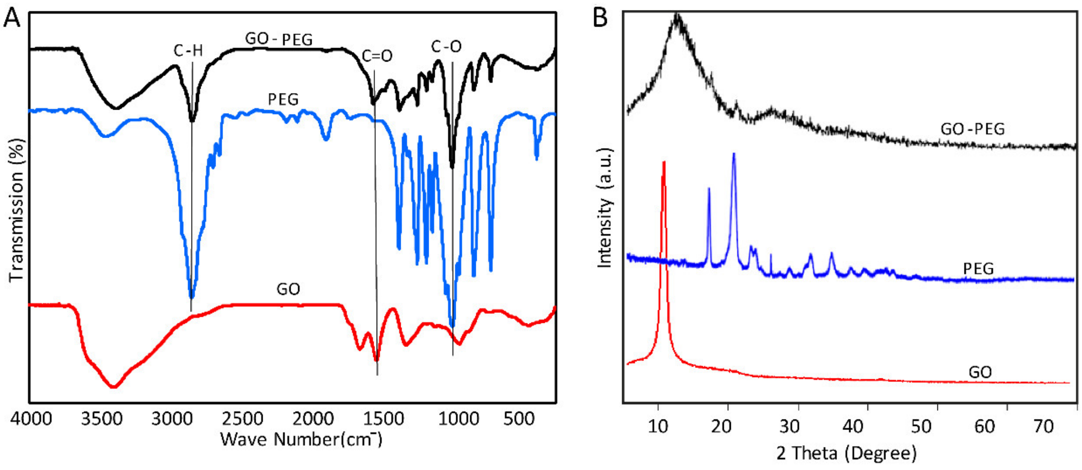

Figure S1. A) FT-IR spectra of GO, PEG, and GO-PEG; **B)** XRD patterns of GO, PEG, and GO-PEG.

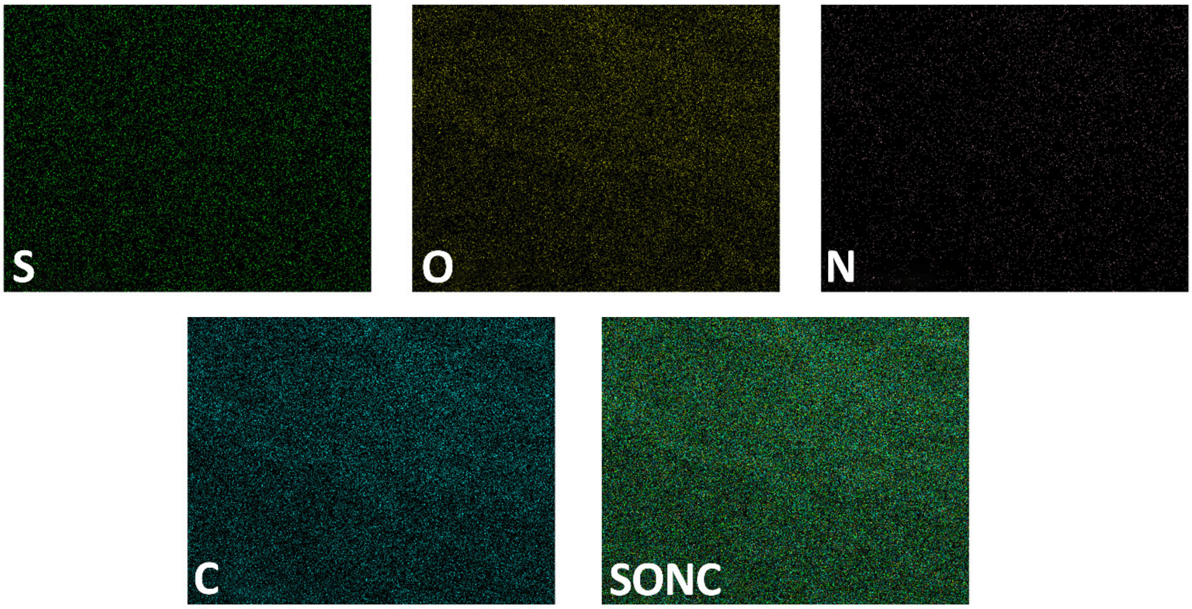

Figure S2. EDX mapping of elements (S, O, N, and C) on the GO-PEG- OXA surface.

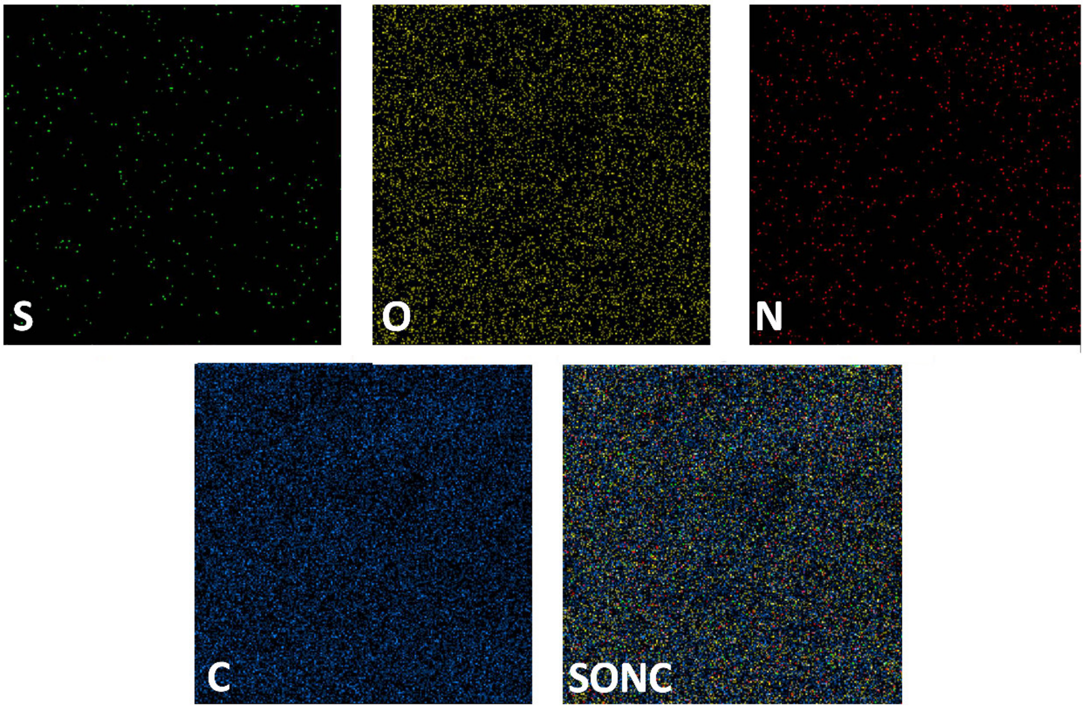

Figure S3. EDX mapping of elements (S, O, N, and C) on the GO-PEG- PEN surface.
